# Rates of Fetal Polydrug Exposures in Methadone-Maintained Pregnancies from a High-Risk Population

**DOI:** 10.1371/journal.pone.0082647

**Published:** 2013-12-02

**Authors:** Kaitlyn Delano, Joey Gareri, Gideon Koren

**Affiliations:** 1 Division of Clinical Pharmacology and Toxicology, The Hospital for Sick Children, Toronto, Ontario, Canada; 2 Department of Pharmacology and Toxicology, University of Toronto, Toronto, Ontario, Canada; 3 Department of Pharmaceutical Sciences, University of Toronto, Toronto, Ontario, Canada; 4 Department of Physiology and Pharmacology, University of Western Ontario, London, Ontario, Canada; Charité, Campus Benjamin Franklin, Germany

## Abstract

Methadone maintenance treatment (MMT) is the standard of care during pregnancy for opioid-dependency, showing efficacy in improving prenatal care and reducing risk of relapse. By design, however, MMT is only intended to prevent withdrawal thus facilitating cognitive behavioural interventions. In order to maximize the benefits of MMT, it is essential that methadone is both properly prescribed and that additional addiction treatment is concurrently administered. This study aims to determine the effectiveness of MMT engagement in high-risk pregnant women in reducing polydrug use by objective laboratory examination of neonatal meconium.

**Patients and Methods:**

Over a 29-month period, the Motherisk Laboratory at the Hospital for Sick Children in Toronto analyzed meconium samples as per request by social services and hospitals for drugs of abuse.

**Results:**

Of the 904 meconium samples received, 273 were tested for methadone with 164 positive and 109 negative for methadone. Almost half of the methadone positive samples (46.34%) were also positive for at least one other opioid compound, which did not differ statistically from the methadone-negative control samples (46.79%; Chi square test, p=0.94). No differences were found between the methadone positive and negative groups in rates of concurrent amphetamines, cocaine, cannabis, and alcohol use indicating a similar risk of polydrug use between pregnant women taking or not taking methadone in this population.

**Discussion:**

The high rates of additional opioid and other drug use in the MMT group, suggest that MMT is failing this population of patients. It is possible that methadone doses during pregnancy are not appropriately adjusted for changes in pharmacokinetic parameters (e.g. blood volume, renal function) during the second and third trimesters. This may result in sub-therapeutic dosing creating withdrawal symptoms leading to additional substance use. Alternatively, these results may be demonstrating a substantial lack in delivery of addiction support services in this vulnerable population.

## Introduction

Over the past decades, there has been an increase in the prevalence of substance abuse among young women, with up to 90% being of reproductive age [1],[2]. This general increase has been paralleled with a marked increase in the prevalence of opioid abuse during pregnancy [3]. The long-acting opioid, methadone, is currently the first line of treatment for opioid dependence for both pregnant and non-pregnant individuals. Methadone maintenance treatment (MMT) provides more stable opioid blood concentrations, resulting in decreased withdrawal symptoms. Prevention of withdrawal is intended to decrease illicit use of other opioids and facilitate addiction treatment interventions. Pregnant women in MMT have been found to take better care of their health and be less likely to relapse during pregnancy when compared to non-MMT, drug-using women [4].

Polydrug use continues to be a serious issue in the drug-using community. The rates of polydrug use in pregnancy are relatively unknown, as maternal self-reports tend to be an unreliable source of information [5]. In contrast to self-reports, the use of biological markers in meconium analysis allow for an objective analysis of drug use during the third, and possibly second trimesters of pregnancy [6],[7]. With the use of meconium analysis for drugs of abuse, the effectiveness of MMT can be estimated using rates of polydrug use during pregnancy as a surrogate marker for clinical progress. 

Meconium is evacuated by neonates over their first few bowel movements; it begins to form in the fetus at around 12 weeks of gestation, when fetal swallowing of amniotic fluid is initiated [8],[9]. Xenobiotics and their metabolites are deposited into meconium by the biliary route or through fetal swallowing [8]. While meconium starts to form at the beginning of the second trimester, evidence suggests that meconium likely reflects third trimester fetal exposures [10]. Meconium is considered a highly sensitive matrix, demonstrating a window of detection far exceeding that afforded by traditional urinalysis and therefore, serves as an optimal matrix for population-based studies examining gestational exposures [11],[12].

The aim of the present study was to examine a population of high-risk, social services-involved women and determine if rates of gestational polydrug use were lower in women on MMT.

## Methods

### Ethics Statement

The study was approved by the Hospital for Sick Children Research Ethics Committee. As approved by the Research Ethics Committee, all results from the Motherisk Laboratory are available for retrospective analysis. The Research Ethics Committee waived written consent from sample donors as database results are anonymized.

### Sample Analysis

This study is a retrospective, observational assessment of meconium toxicology performed on samples referred by physicians, primarily at the request of social services, over a 29-month period. Meconium samples were shipped frozen from sites of collection to the Motherisk Laboratory at the Hospital for Sick Children and stored at -80°C until analysis. 

Briefly, 0.300 g of meconium was thawed at room temperature, homogenized and transferred into a 13mL test tube (Sarstedt; Montreal, QC). One mL of methanol was added to each sample, vortexed for 30 seconds then centrifuged for 15 min at 3,500 rpm at room temperature. The supernatant for each sample was then decanted into a 5mL test tube. Samples were then screened by ELISA (Immunalysis, Pomona CA) for the following drugs and metabolites: cocaine, benzoylecgonine, opiates (including codeine, morphine, 6-monoacetylmorphine, hydrocodone and hydromorphone), oxycodone (with cross-reactivity to oxymorphone), amphetamine, methamphetamine, delta-9-tetrahydroxycannabinol (THC), benzodiazepines, barbiturates, meperidine, and methadone. One hundred μL aliquots of the methanol extract are taken for each individual ELISA test and 400μL of phosphate buffered saline (PBS) is added. Samples are then added to the ELISA plate, 100μL of enzyme conjugate added, then incubated for 60 min at room temperature in the dark. Wells are then washed with ddH_2_O and 100μL of substrate reagent is added to each well and then incubated again for 30 min at room temperature in the dark. Stop solution is added and absorbance is then measured at a dual wavelength of 450nm and 650nm using a SUNRISE Absorbance Reader (Tecan Systems Inc, San Jose CA). Samples are then deemed positive or negative, if the absorbance is below or above the cut-off standard respectively.

THC analysis was performed on ELISA only; all other screen-positive ELISA samples were subsequently confirmed through GC-MS analysis using previously published methods for drugs of abuse and alcohol metabolites [13],[14]. The drugs of abuse GC-MS method is capable of simultaneously detecting and quantifying cocaine, benzoylecgonine, norcocaine, cocaethylene, 6-monoacetylmorphine, morphine, codeine, oxycodone, oxymorphone, hydrocodone, hydromorphone, meperidine, methadone, amphetamine, methamphetamine, 3,4-methylenedioxymethamphetamine, and methylenedioxyamphetamine. Deuterated internal standards of the 17 analytes and 20% methoxiamine solution is added to an aliquot of the previously described methanol extract from each sample, vortexed and incubated at room temperature for 1 hour. Two mL of 0.1M phosphate solution is then added to each sample, vortexed and incubated for 15 min at room temperature. Samples then undergo automated SPE extraction (Gilson ASPEC GX-274 Automated SPE extractor; Middleton, WI, USA), transferred to a 10mL SPME vial (Supelco; Bellefonte, PA) and then dried under nitrogen at 35°C. Once dried, 20 μL of derivatizing mixture (BSTFA and MSTFA 3:1) is added and then samples are analyzed via GC-MS on a Shimadzu QP2010 Plus (Shimadzu Scientific Inc; Columbia, MD).

For quality control procedures, quality control samples are analyzed after every 20 samples. These quality control samples are blank meconium specimens, which are spiked with a known amount of the tested compounds. Any discordance between ELISA and GC-MS analyses results in reanalysis of the sample.

### Data Analysis

Meconium results were dichotomized into two groups, positive and negative for methadone. Number and proportions of positive drug classes (cocaine, amphetamines, opioids, cannabinoids, benzodiazepines and alcohol) were calculated. As some of the compounds tested are both metabolites of a drug of abuse and drugs of abuse themselves, the source of these compounds cannot be determined, thus requiring the calculation of minimum and maximum number of positive drugs. To further elaborate, morphine is itself a drug of abuse as well as a metabolite of both codeine and heroin. Hydromorphone and oxymorphone are also drugs of abuse but also metabolites of hydrocodone and morphine, and oxycodone respectively. Finally, amphetamine is both a metabolite of methamphetamine and a drug of abuse itself. This apparent overlap must be taken into consideration when determining the number of positive drugs detected in the meconium samples.

For statistical analyses, Chi Square and Fisher’s Exact tests were performed, where appropriate, to compare the rates of positivity of individual drugs and drug classes between methadone positive and methadone negative samples. Mann Whitney U test was performed to compare the mean number of positive drugs overall and mean number of positive opioids between the two groups. All statistical tests were performed using IBM SPSS Statistics 20.0. 

## Results

Of the 273 meconium samples analyzed for methadone, 164 were found to be positive and 109 were negative. Fifty-eight (35.37%) of methadone-positive samples were positive, at minimum, for one additional opioid and 18 (10.98%) were positive for 2 additional opioids. In regards to maximum number of positive opioids, 39 (23.78%), 19 (11.59%), 14 (8.54%), 3 (1.83%) and 1 (0.61%) were positive for 1, 2, 3, 4 and 5 additional opioids respectively.

No statistically significant differences were found with regards to proportions of illicit drug use between the methadone-positive and negative groups for any drug class (Table 1); although a trend towards higher rates of amphetamine (drug) and benzodiazepine (class) use was noted (Table 2). As well, no statistical difference was found for mean minimum number of positive drugs (methadone positive 1.293, SD = 0.997; methadone negative 1.431, SD= 1.141; p=0.477). Also, no statistical difference was found for mean maximum number of positive drugs (methadone positive 1.567, SD = 1.348; methadone negative 1.716, SD = 1.441; p=0.451).

**Table 1:** Rates of positivity for six drug classes.

	Rate of Positivity (%) (n)		
Drug Class	Methadone Positive (N=164)	Methadone Negative (N=109)	P value
Amphetamines	4.27 (7)	2.75 (3)	0.744**^&^**
Cocaine	30.49 (50)	26.61 (29)	0.488**^^^**
Opioids	46.34 (76)	46.79 (51)	0.942**^^^**
Cannabinoids^*^	55.10 (54)	52.17 (48)	0.686**^^^**
Benzodiazepines**^#^**	14.29 (4)	3.23 (2)	0.073**^&^**
Alcohol**^+^**	5.88 (4)	6.67 (4)	1**^&^**

**^^^**Pearson Chi-square test performed; **^&^**Fisher’s Exact test performed; *Methadone positive n=98, Methadone negative n=92; **^#^**Methadone positive n=28, Methadone negative n=62; **^+^**Methadone positive n=68, Methadone negative n=60

**Table 2:** Rates of positivity for individual compounds.

	Rate of Positivity (%) (n)		
Compound	Methadone Positive	Methadone negative	P value
Amphetamine^*^	5.31 (6)	1.09 (1)	0.132**^&^**
Methamphetamine**^#^**	3.57 (4)	3.26 (3)	1**^&^**
MDMA^$^	0	2.17 (2)	0.204**^&^**
Meperidine^%^	4.55 (1)	1.09 (1)	0.440**^&^**
Cocaine**^=^**	23.46 (38)	21.30 (23)	0.677**^^^**
Cocaethylene**^+^**	2.47 (4)	0.94 (1)	0.651**^&^**
Benzoylecgonine**^*a*^**	30.86 (50)	27.78 (30)	0.586**^^^**
Norcocaine**^*a*^**	12.30 (21)	14.81 (16)	0.665**^^^**
Codeine**^*a*^**	11.73 (19)	16.67 (18)	0.248**^^^**
Morphine**^*a*^**	29.63 (48)	20.37 (22)	0.089**^^^**
6-MAM**^*a*^**	0.62 (1)	0.93 (1)	1**^&^**
Hydromorphone**^*a*^**	11.73 (19)	11.11 (12)	0.876**^^^**
Oxycodone**^*a*^**	17.28 (28)	22.22 (24)	0.313**^^^**
Oxymorphone**^*a*^**	12.96 (21)	16.67 (18)	0.396**^^^**

**^^^**Pearson Chi-square test performed; **^&^**Fisher’s Exact test performed; *Methadone positive n=113, Methadone negative n=92; **^#^**Methadone positive n=112, Methadone negative n=92; **^$^**Methadone positive n=111, Methadone negative n=92; **^%^**Methadone positive n=22, Methadone negative n=66; **^=^**Methadone positive n=162, Methadone negative n=108; **^+^**Methadone positive n=162, Methadone negative n=106; **^*a*^**Methadone positive n=162, Methadone negative n=108

When looking specifically at opioids, no statistical difference was found for either mean minimum or maximum number of positive opioids (min: methadone positive 0.580, SD = 0.685; methadone negative 0.602, SD = 0.723; p=0.907; max: methadone positive 0.840, SD=1.114; methadone negative 0.880, SD=1.174; p=0.913). 

## Discussion

Individuals are expected to be engaged in MMT with the clear clinical goal of replacing the need for other opioids. While in most programs, routine urine tests are conducted to verify that indeed this is the case; sample tempering is a major issue [15],[16]. Our results, based on meconium analysis, which yields an integral of fetal exposure to drugs over at least the last trimester of pregnancy, indicate that at least one third of pregnant women on MMT continue to use at least one additional opioid narcotic. When comparing them to meconium with no detected methadone, indicating that the mother was not on MMT, no significant difference was found in proportion of opioid or other illicit drug use between the methadone positive and negative meconium samples, indicating similar patterns of substance use during late pregnancy. This alarming finding of a lack of an apparent decrease in opioid use for those positive for methadone means ineffective MMT, and increased burden of illicit drug exposure to the fetus. 

With such a large proportion of the methadone positive meconium samples also being positive for at least one other opioid, the efficacy and effectiveness of methadone is brought into question. Changes in clearance rate and volume of distribution during pregnancy may affect the efficacy of methadone. Wolff et al. (2005) found that weight-adjusted methadone clearance increased between the first and third trimesters [17]. Dickmann and Isoherranen (2012) observed increased CYP2B6 activity in human hepatocytes due to estradiol induction, which would increase methadone metabolism via CYP2B6 [18]. Additionally, Jarvis et al (2008) determined, in a population of methadone dependent pregnant females, that the half-life of methadone was almost one half that of non-pregnant methadone users [19]. Absorption of methadone may also be affected during pregnancy, as gastric acid secretions decrease, inhibiting gastrointestinal absorption [19]. Overall, these pharmacokinetic changes may render the prescribed dose of methadone subtherapeutic. The unstable exposure to opioids in utero may induce fetal stress, increase the risk of abortion and preterm birth [20].

Polydrug use was found in both methadone positive and negative cohorts with cannabinoids, opioids and cocaine as the most common drug classes detected in the samples for both groups. This highlights the numerous compounds the fetus is exposed to, and the increased risk of fetal stress and adverse outcomes. 

The population assessed in this study is high-risk; almost all women whose neonates were tested are actively involved with social service organizations and demonstrate ongoing child protection concerns. This study assumes that methadone present in meconium is the result of MMT, however, methadone is used illicitly as well and it should be considered that a proportion of the methadone-positive group may not be actively engaged in MMT. While the characteristics of the study population limit the generalizability of these findings to the MMT population at large, the lack of effectiveness of MMT in this particular population underscores current limitations in the delivery of effective addiction treatment services to this highly vulnerable group. Despite substantial mobilization of government resources to this population, they remain under-served in meeting their addiction treatment needs. While comprehensive strategies for addressing substance addiction in pregnancy have been extensively reported in the literature [21],[22],[23],[24]; these data demonstrate that the current model of MMT delivery does not address the addiction treatment needs of women who have already been identified as high-needs.

This study demonstrates a need for closer clinical monitoring of pregnant women in MMT to ensure that treatment is effective in preventing maternal or neonatal withdrawal symptoms, continued opioid or polydrug use, and adverse neonatal outcomes. The high rate of polydrug use in the methadone-positive cohort demonstrates that the current level of care provided to these women is ineffective and should be augmented with consideration given to: i) therapeutic drug monitoring of methadone to evaluate necessity for dose increases due to altered pharmacokinetics in pregnancy, ii) alternative therapies, such as buprenorphine, that can provide a more stable maintenance treatment regime and result in potentially better neonatal outcomes [25], iii) ensuring accessibility of complementary non-pharmacological interventions (e.g. cognitive behavioural therapy, etc.) that are an established requirement to successful addiction treatment.

## References

[1] Kuczkowski KM (2007) The effects of drug abuse on pregnancy. Curr Opin Obstet Gynecol 19: 578-585. doi:10.1097/GCO.0b013e3282f1bf17. PubMed: 18007137. 18007137

[2] Keegan J, Parva M, Finnegan M, Gerson A, Belden M (2010) Addiction in pregnancy. J Addict Dis 29: 175-191. doi:10.1080/10550881003684723. PubMed: 20407975. 20407975

[3] Kelly L, Dooley J, Cromarty H, Minty B, Morgan A et al. (2011) Narcotic-exposed neonates in a First Nations population in northwestern Ontario: incidence and implications. Can Fam Physician 57: e441-e447. PubMed: 22084474. 22084474 PMC3215628

[4] Kandall SR, Albin S, Gartner LM, Lee KS, Eidelman A et al. (1977) The narcotic-dependent mother: fetal and neonatal consequences. Early Hum Dev 1: 159-169. doi:10.1016/0378-3782(77)90017-2. PubMed: 617308. 617308

[5] Bessa MA, Mitsuhiro SS, Chalem E, Barros MM, Guinsburg R et al. (2010) Underreporting of use of cocaine and marijuana during the third trimester of gestation among pregnant adolescents. Addict Behav 35: 266-269. doi:10.1016/j.addbeh.2009.10.007. PubMed: 19896774. 19896774

[6] Gray TR, Eiden RD, Leonard KE, Connors G, Shisler S et al. (2010) Nicotine and metabolites in meconium as evidence of maternal cigarette smoking during pregnancy and predictors of neonatal growth deficits. Nicotine Tob Res 12: 658-664. doi:10.1093/ntr/ntq068. PubMed: 20427459. 20427459 PMC2878732

[7] Bar-Oz B, Klein J, Karaskov T, Koren G (2003) Comparison of meconium and neonatal hair analysis for detection of gestational exposure to drugs of abuse. Arch Dis Child Fetal Neonatal Ed 88: F98-F100. doi:10.1136/fn.88.2.F98. PubMed: 12598495. 12598495 PMC1721515

[8] Kwong TC, Ryan RM (1997) Detection of intrauterine illicit drug exposure by newborn drug testing. National Academy of Clinical. Biochemistry - Clin Chem 43: 235-242. 8990259

[9] Miller V, Holzel A (1974) Growth and development of endodermal structures. Scientific foundations in paediatrics. Philadelphia: WB Saunders pp. 281-296.

[10] Kacinko SL, Jones HE, Johnson RE, Choo RE, Huestis MA (2008) Correlations of maternal buprenorphine dose, buprenorphine, and metabolite concentrations in meconium with neonatal outcomes. Clin Pharmacol Ther 84: 604-612. doi:10.1038/clpt.2008.156. PubMed: 18701886. 18701886 PMC2714885

[11] Moller M, Gareri J, Koren G (2010) A review of substance abuse monitoring in a social services context: a primer for child protection workers. Can J Clin Pharmacol 17: e177-e193. PubMed: 20436210. 20436210

[12] Koren G, Hutson J, Gareri J (2008) Novel methods for the detection of drug and alcohol exposure during pregnancy: implications for maternal and child health. Clin Pharmacol Ther 83: 631-634. doi:10.1038/sj.clpt.6100506. PubMed: 18288086. 18288086

[13] Aleksa K, Walasek P, Fulga N, Kapur B, Gareri J et al. (2012) Simultaneous detection of seventeen drugs of abuse and metabolites in hair using solid phase micro extraction (SPME) with GC/MS. Forensic Sci Int 218: 31-36. doi:10.1016/j.forsciint.2011.10.002. PubMed: 22047752. 22047752

[14] Hutson JR, Rao C, Fulga N, Aleksa K, Koren G (2011) An improved method for rapidly quantifying fatty acid ethyl esters in meconium suitable for prenatal alcohol screening. Alcohol 45: 193-199. doi:10.1016/j.alcohol.2010.07.005. PubMed: 20705417. 20705417

[15] Clancy Z, O’Connell K, Couto J (2013) The use of urine drug monitoring in chronic opioid therapy: an analysis of current clinician behavior. J Opioid Manag 9: 121-127. doi:10.1080/17445647.2012.758423. PubMed: 23709321. 23709321

[16] Jaffee WB, Trucco E, Levy S, Weiss RD (2007) Is this urine really negative? A systematic review of tampering methods in urine drug screening and testing. J Subst Abuse Treat 33: 33-42. doi:10.1016/j.jsat.2006.11.008. PubMed: 17588487. 17588487

[17] Wolff K, Boys A, Rostami-Hodjegan A, Hay A, Raistrick D (2005) Changes to methadone clearance during pregnancy. Eur J Clin Pharmacol 61: 763-768. doi:10.1007/s00228-005-0035-5. PubMed: 16261362. 16261362

[18] Dickmann LJ, Isoherranen N (2013) Quantitative prediction of CYP2B6 induction by estradiol during pregnancy: potential explanation for increased methadone clearance during pregnancy. Drug Metab Dispos 41: 270-274. doi:10.1124/dmd.112.047118. PubMed: 22815312. 22815312

[19] Jarvis MA, Wu-Pong S, Kniseley JS, Schnoll SH (1999) Alterations in methadone metabolism during late pregnancy. J Addict Dis 18: 51-61. doi:10.1300/J069v18n02_05. PubMed: 10631963. 10631963

[20] McCarthy JJ (2012) Intrauterine abstinence syndrome (IAS) during buprenorphine inductions and methadone tapers: can we assure the safety of the fetus? J Matern Fetal Neonatal Med 25: 109-112. doi:10.3109/14767058.2011.600371. PubMed: 21867403. 21867403

[21] Finnegan LP, Connaughton JF, Emich JP, Wieland WF (1971) Comprehensive care of the pregnant addict and its effect on maternal and infant outcome. ContemP. Drug Probs 1: 795.

[22] Pepler DJ, Moore TE, Motz M, Leslie M (2002) Breaking the cycle: The evaluation report (1995-2000). Toronto. Health Canada.

[23] Poole N (2000) Evaluation report of the Sheway Project for high-risk pregnant and parenting women. British Columbia Centre of Excellence for Women’s Health, Sheway Project, Vancouver, BC.

[24] Loudenburg R, Leonardson GR (2003) A multifaceted intervention strategy for reducing substance use in high-risk women. Neurotoxicol Teratol 25: 737-744. doi:10.1016/j.ntt.2003.07.009. PubMed: 14624974. 14624974

[25] Welle-Strand GK, Skurtveit S, Jones HE, Waal H, Bakstad B et al. (2013) Neonatal outcomes following in utero exposure to methadone or buprenorphine: a National Cohort Study of opioid-agonist treatment of Pregnant Women in Norway from 1996 to 2009. Drug Alcohol Depend 127: 200-206. doi:10.1016/j.drugalcdep.2012.07.001. PubMed: 22841456. 22841456

